# Reliable Diameter Control of Carbon Nanotube Nanobundles Using Withdrawal Velocity

**DOI:** 10.1186/s11671-016-1600-9

**Published:** 2016-09-01

**Authors:** Jung Hwal Shin, Kanghyun Kim, Taechang An, WooSeok Choi, Geunbae Lim

**Affiliations:** 1 Department of Mechanical Engineering, Pohang University of Science and Technology (POSTECH), 77 Cheongam-Ro. Nam-Gu, Pohang, Gyeongsangbuk-do 790-784 Republic of Korea; 2 Department of Mechanical Design Engineering, Andong National University, Andong, Gyungbuk 760-749 Republic of Korea; 3 Department of Mechanical Engineering, Korea National University of Transportation, 50 Daehak-Ro, Chungju, Chungcheongbuk-do, Chungcheongbuk-do 380-702 Republic of Korea

**Keywords:** Carbon nanotube (CNT), CNT nanobundle, Capillary force, van der Waals force, Withdrawal velocity, Attachment efficiency

## Abstract

Carbon nanotube (CNT) nanobundles are widely used in nanoscale imaging, fabrication, and electrochemical and biological sensing. The diameter of CNT nanobundles should be controlled precisely, because it is an important factor in determining electrode performance. Here, we fabricated CNT nanobundles on tungsten tips using dielectrophoresis (DEP) force and controlled their diameters by varying the withdrawal velocity of the tungsten tips. Withdrawal velocity pulling away from the liquid–air interface could be an important, reliable parameter to control the diameter of CNT nanobundles. The withdrawal velocity was controlled automatically and precisely with a one-dimensional motorized stage. The effect of the withdrawal velocity on the diameter of CNT nanobundles was analyzed theoretically and compared with the experimental results. Based on the attachment efficiency, the withdrawal velocity is inversely proportional to the diameter of the CNT nanobundles; this has been demonstrated experimentally. Control of the withdrawal velocity will play an important role in fabricating CNT nanobundles using DEP phenomena.

## Background

Carbon nanotubes (CNTs) have intrinsically large surface areas (700–1000 m^2^ g^−1^), chemical stability, and excellent mechanical and electrical properties, including an extremely high conductance and aspect ratio [1]. For these reasons, CNTs are widely used as electrode materials for improving biocompatibility and additional functional properties [2–5]. Based on these favorable characteristics, the use of CNTs for biosensor applications, in the form of modified electrode [6–8], polymer-CNT composites [9], and CNT membranes [10] has received much attention.

CNT nanobundles have contributed to the development of nanotechnology providing many advantages in nanoscale imaging, fabrication, and electrochemical and biological sensing due to their superior geometric, electronic, chemical, and mechanical properties [11–16]. Various methods have been developed to attach or directly grow CNTs on the apex of micro-tips, such as a tungsten tip or an atomic force microscope (AFM) tip [17–23]. However generally, these techniques are time-consuming and not viable as commercial techniques. To overcome these limitations, new fabrication methods for attaching CNTs on a tip apex have been introduced using dielectrophoresis (DEP) phenomena [24–26] which are non-invasive, non-destructive, and active methods for the manipulation, alignment, and separation of particles at the microscale [27–29].

Generally, it is important to control the diameter of the electrode because it can determine electrode performance. Parameters such as withdrawal velocity, applied voltage and frequency, and CNT concentration are important factors in determining the electrode diameter. However, few studies exist regarding control of the diameter of CNT nanobundles attached to a tip. Kim et al. performed a numerical simulation of CNT nanobundles attached to an AFM tip, excluding any finite-size effect of CNTs on the DEP force and torque [30]. Wei et al. showed that the diameter of CNT nanobundles grew asymptotically as the voltage increased from 5 to 19 V, and tip wetting and geometry played a key role using eight-type scanning probe microscopy (SPM) probes [31]. However, no previous study has reported the effects of the withdrawal velocity, which is a reliable parameter to control the diameter of CNT nanobundles.

In this study, we fabricated CNT nanobundles using DEP phenomena. The withdrawal velocity of a tungsten tip was controlled precisely using a one-dimensional (1-D) motorized stage, and the fabricated CNT nanobundles were observed by scanning electron microscope (SEM). The effect of the withdrawal velocity on the diameter of CNT nanobundles was investigated theoretically and experimentally.

## Methods

### Preparation of Sharpened Tungsten Tips

**Fig. 1:**
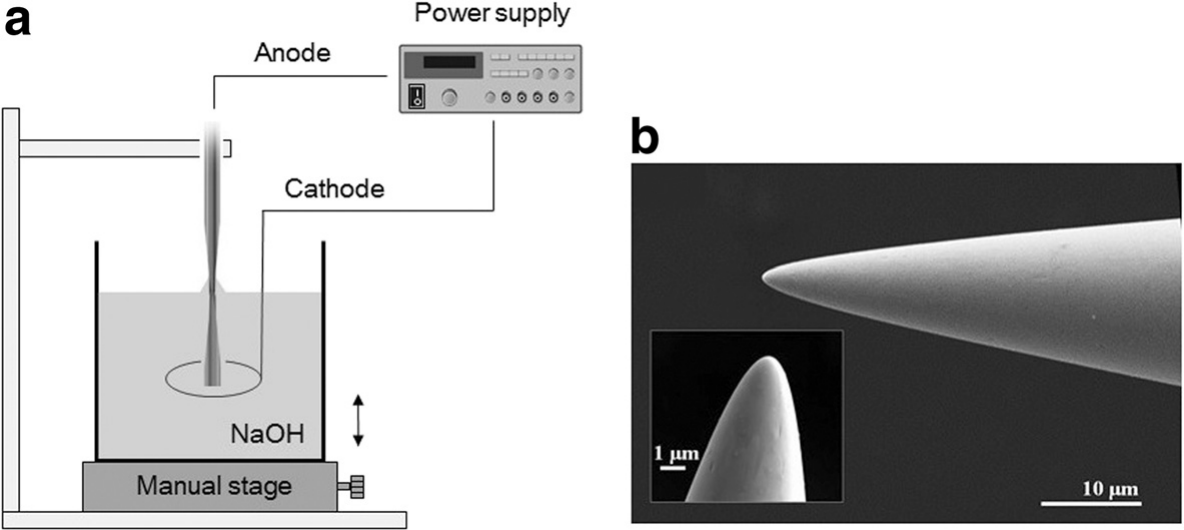
Preparation of a sharpened tungsten tip. **a** Schematic diagram of process for a sharpened tungsten tip using electrochemical etching. **b** Scanning electron microscopy (SEM) image of the sharpened tungsten tip obtained by electrochemical etching

Sharpened tungsten tips were prepared with an electrochemical etching method which is one of the easiest and fastest methods to obtain cheap and reliable metallic probes. Figure 1a shows a schematic diagram of the electrochemical etching setup for preparing a sharpened tungsten tip. A highly pure tungsten wire (300 μm diameter, 99.9 % purity), purchased from Korea Tungsten (Korea), was mounted on a holder. The tungsten wire (anode) and a counter electrode (nickel wire, cathode) were immersed in a 2-M NaOH solution, and a reservoir containing the solution was placed on a 1-D manual stage. Electrochemical etching occurred at the air-electrolyte interface when a direct current (DC) voltage was applied (>5 V) between the tungsten wire and the counter electrode. The corresponding electrochemical reactions on the tungsten tip and the counter electrode proceeded according to the following equations:

1 $$ \begin{array}{l}\mathrm{Cathode}:\ 6{\mathrm{H}}_2\mathrm{O} + 6{\mathrm{e}}^{-}\to\ 3{\mathrm{H}}_2\left(\mathrm{g}\right) + 6{\mathrm{OH}}^{-}\\ {}\mathrm{Anode}:\ \mathrm{W}\ \left(\mathrm{s}\right) + 8{\mathrm{OH}}^{-}\to\ {{\mathrm{WO}}_4}^{2-} + 4{\mathrm{H}}_2\mathrm{O} + 6{\mathrm{e}}^{-}\\ {}\mathrm{Overall}:\ \mathrm{W}\ \left(\mathrm{s}\right) + 2{\mathrm{OH}}^{-} + 2{\mathrm{H}}_2\mathrm{O}\ \to\ {{\mathrm{WO}}_4}^{2-} + 3{\mathrm{H}}_2\left(\mathrm{g}\right)\end{array} $$

To obtain a smooth and sharp tip apex without the “neck-in” phenomenon, the tungsten wire was steadily shuttled up and down during the electrochemical reaction. Figure 1b shows a SEM image of the fabricated sharpened tungsten tip obtained by this process.

### Preparation of the CNT Suspension

**Fig. 2:**
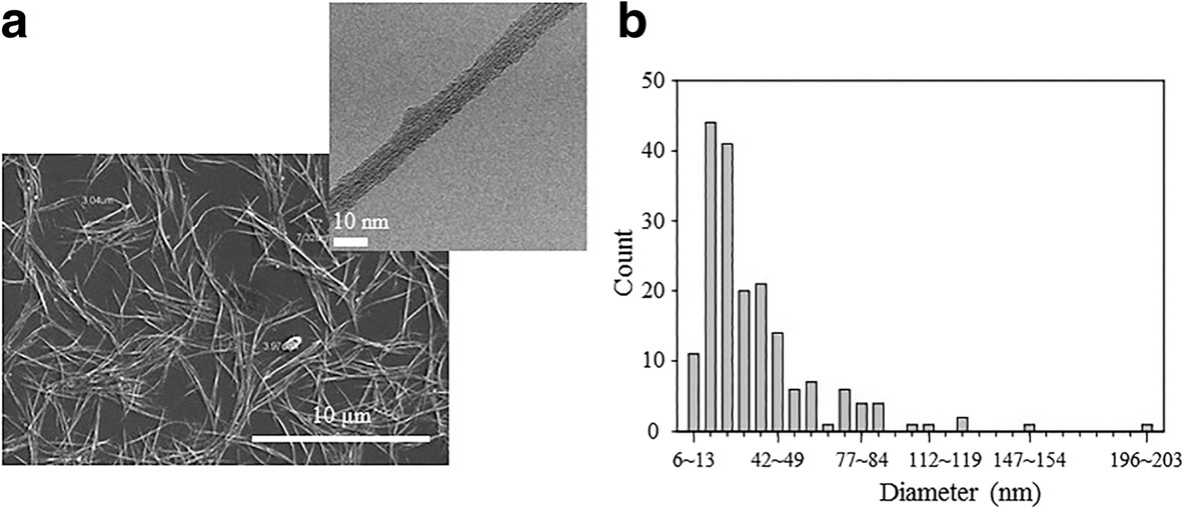
The well-dispersed CNT suspension. **a** Scanning electron microscopy (SEM) and transmission electron microscopy (TEM) images of the dispersed SWNTs and a single carbon nanotube (CNT) strand. **b** CNTs diameter distribution

A CNT suspension was prepared by a strong acid treatment. First, single-walled nanotubes (SWNTs) of 5 mg (Hanwha Nanotech, Seoul, South Korea) were suspended in a concentrated H_2_SO_4_:HNO_3_ (50 mL, 3:1 *v*/*v*) solution and sonicated in an ultrasonication bath for 3 h. During the acid treatment, catalyst particles and amorphous carbon particles were removed. Through oxidizing under the strong acid conditions, SWNTs were functionalized with carboxyl groups (COOH^−^) [32]. Second for neutralizing the solution, a large amount of DI water was mixed and the acid solution was removed by vacuum filtration using a 0.22-μm filter (GVWP, Millipore), repeatedly. After the pH of the solution reached 7, the undispersed CNT suspension was removed by centrifugation (12,000 rpm, 10 min). Finally, a well-dispersed CNT suspension was prepared and the length of CNTs was about 3~7 μm (Fig. 2a). Figure 2b shows the CNT diameter distribution, and the diameter of CNTs was about 6~49 nm (82 % of the total CNTs diameter). A CNT concentration has a significant effect on the CNT nanobundle diameter. To fix “CNT concentration” parameter, all experiments were carried out using one CNT suspension.

### Experimental Setup for Fabricating CNT Nanobundles

**Fig. 3:**
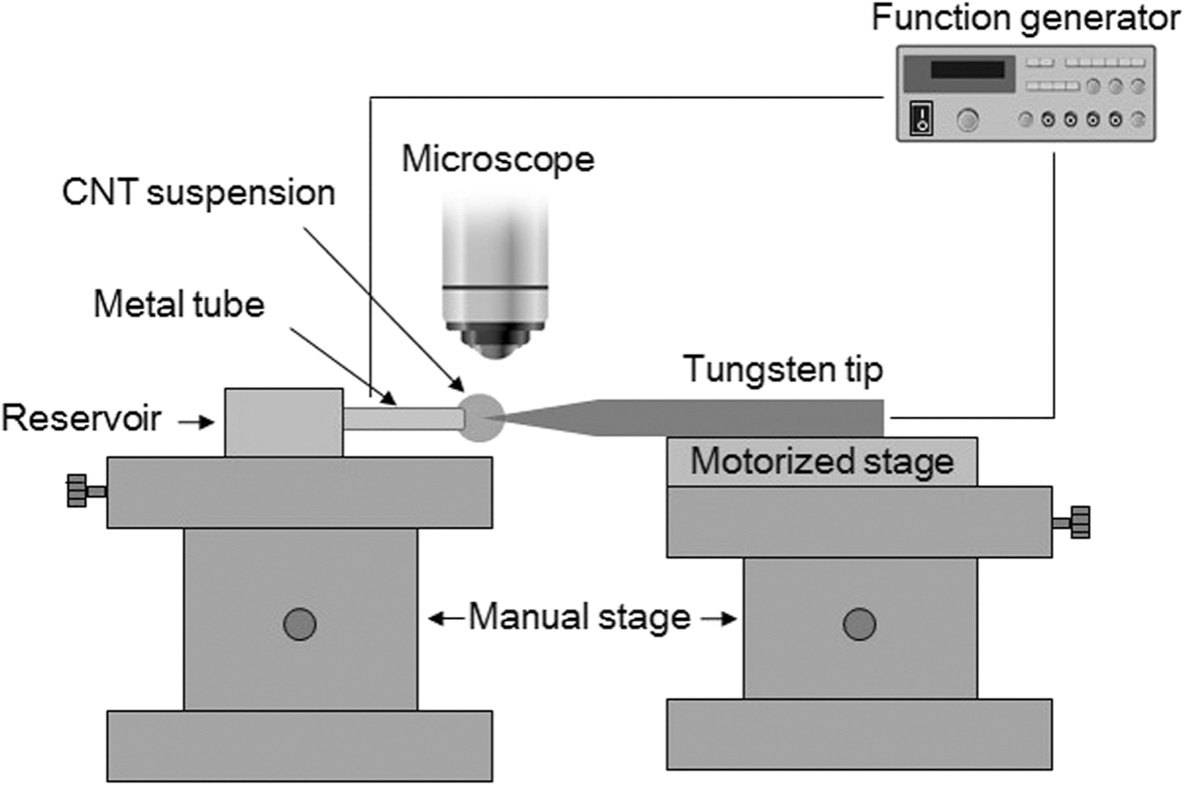
Schematic diagram of the experimental setup for fabricating a CNT nanobundle on a tungsten tip

Figure 3 shows the experimental setup for fabricating CNT nanobundles on tungsten tips. The setup consisted of a metal tube, a manual stage, a 1-D motorized stage (PI, Compact Micro-Translation series), a sharpened tungsten tip, an optical microscopy setup, and a function generator (Tektronix, AFG3101). The CNT suspension was placed in a reservoir connected to a metal tube with a 500-μm inner diameter. A tungsten tip was mounted on a holder, which was attached to the 1-D motorized stage. A function generator was connected to the metal tube and the tungsten tip. The 1-D motorized stage and function generator were connected to a PC and controlled with LabVIEW software.

**Fig. 4:**
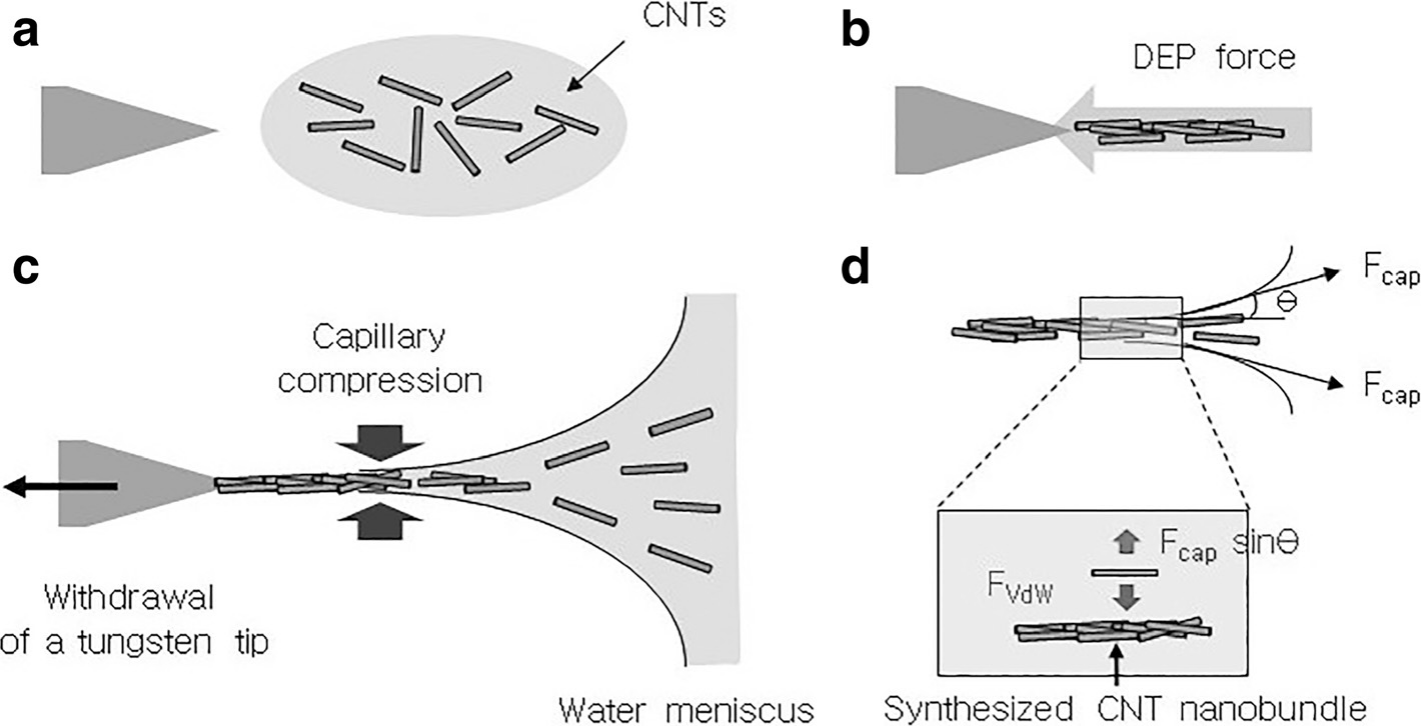
Mechanism of individual CNT assembly into a CNT nanobundle on a tungsten tip under an asymmetric alternating current (AC) electric field. **a** CNTs were are dispersed in a CNT suspension. **b** The dispersed CNTs migrate to around the tip apex due to the non-uniform electric field generated by a positive dielectrophoresis (DEP) force. **c** When the tungsten tip is pulled out from the CNT suspension, capillary compression at the liquid–air interface makes thin wires. **d** The applied forces acting on the CNTs at the liquid–air interface

The fabrication process for the CNT nanobundle on a tungsten tip is illustrated in Fig. 4. Due to the small diameter of the metal tube and surface tension, the CNT suspension stayed on the metal tube with a convex meniscus. The tungsten tip was slightly submerged in the CNT suspension, and an alternating current (AC) electric field was applied between the tungsten tip and the metal tube (Fig. 4a). When the AC electric field was applied, a non-uniform electric field was generated in the CNT suspension because the metal tube had a relatively large area while the tungsten tip was immersed in the CNT suspension only at the tip apex. The dispersed CNTs migrated to around the tungsten tip apex where a high electric field gradient was generated due to a positive DEP force (Fig. 4b). Some CNTs attached to the tungsten tip apex due to van der Waals forces and became the new outermost surface of the electrode where the next CNTs were precipitated. The CNT nanobundle grew continuously with repeated CNT migration and precipitation (Fig. 4c). When the tungsten tip was pulled away from the liquid–air interface, the capillary and van der Waals forces adjusted the CNT alignment and diameter of the CNT nanobundle (Fig. 4d). The applied forces acting on the CNTs in the liquid–air interface are analyzed in the next paragraph.

## Results and Discussion

### The Force Acting on CNTs at the Liquid–Air Interface

Adhesion force of CNTs to a synthesized CNT nanobundle is mostly determined by van der Waals interactions. The van der Waals force per unit length (*F*_vdW_) between the cylindrical tubes such as the CNTs can be expressed as follows:

2 $$ {F}_{\mathrm{vdW}}=\frac{A\sqrt{d/2}}{12\sqrt{2}{x}^{5/2}}, $$

where *A*, *d*, and *x* are the Hamaker constant, the diameter of the CNTs, and the space between the CNTs, respectively. Generally, the Hamaker constant representing the attraction force between molecules, SWNTs in this case, is assumed to be ~0.84 × 10^−19^ J [33, 34]. Thus, the van der Waals force is about *F*_vdW_ = 168 nN/μm, assuming that the diameter of the CNTs is about 10 nm (Fig. 2) and the interlayer spacing of the CNTs is *x* = 0.34  nm[35].

When CNTs are removed from a liquid–air interface, they experience a shear force (~capillary force, *F*_cap_) caused by the surface tension of a liquid. If this force acting on the CNTs is larger than the adhesion force between the CNTs and CNT nanobundle, CNTs cannot escape from the suspension and are finally washed out. Dujardin et al. found that the capillary force acting on CNTs was about 130~170 mN/m at a water-based interface with the surface tension of which was 70 mN/m at room temperature [36]. When the diameter of the CNTs is about 20 nm (Fig. 2), the maximum capillary force acting on CNTs is about *F*_cap_ = 10 nN. This value is much smaller than the adhesion force between the CNTs and a synthesized CNT nanobundle (*F*_vdW_ = 200 nN/μm). Therefore, most of the CNTs were attached to a synthesized CNT nanobundle and were used to constitute a CNT nanobundle.

### Effect of Withdrawal Velocity on CNT Nanobundle Diameter

Under the same conditions (electrode size, electric field, and concentration of CNT suspension), the same amount of CNTs gather in the same area of the meniscus. Generally, one of the most important parameters to determine the diameter of CNT nanobundles is the attachment efficiency. The attachment efficiency is the ratio of particle numbers striking the collector to that around the collector and proportional to the diameter of the collector [37]. In this study, a synthesized CNT nanobundle (the tip of the CNT nanobundle being synthesized) acted as a collector.

**Fig. 5:**
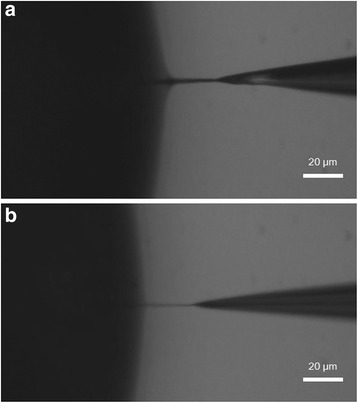
Optical microscopic images. **a** *v*_w_ = 1  μm/s. **b** *v*_w_ = 10  μm/s(AC electric field 5*V*_pp_ and 1 MHz)

**Fig. 6:**
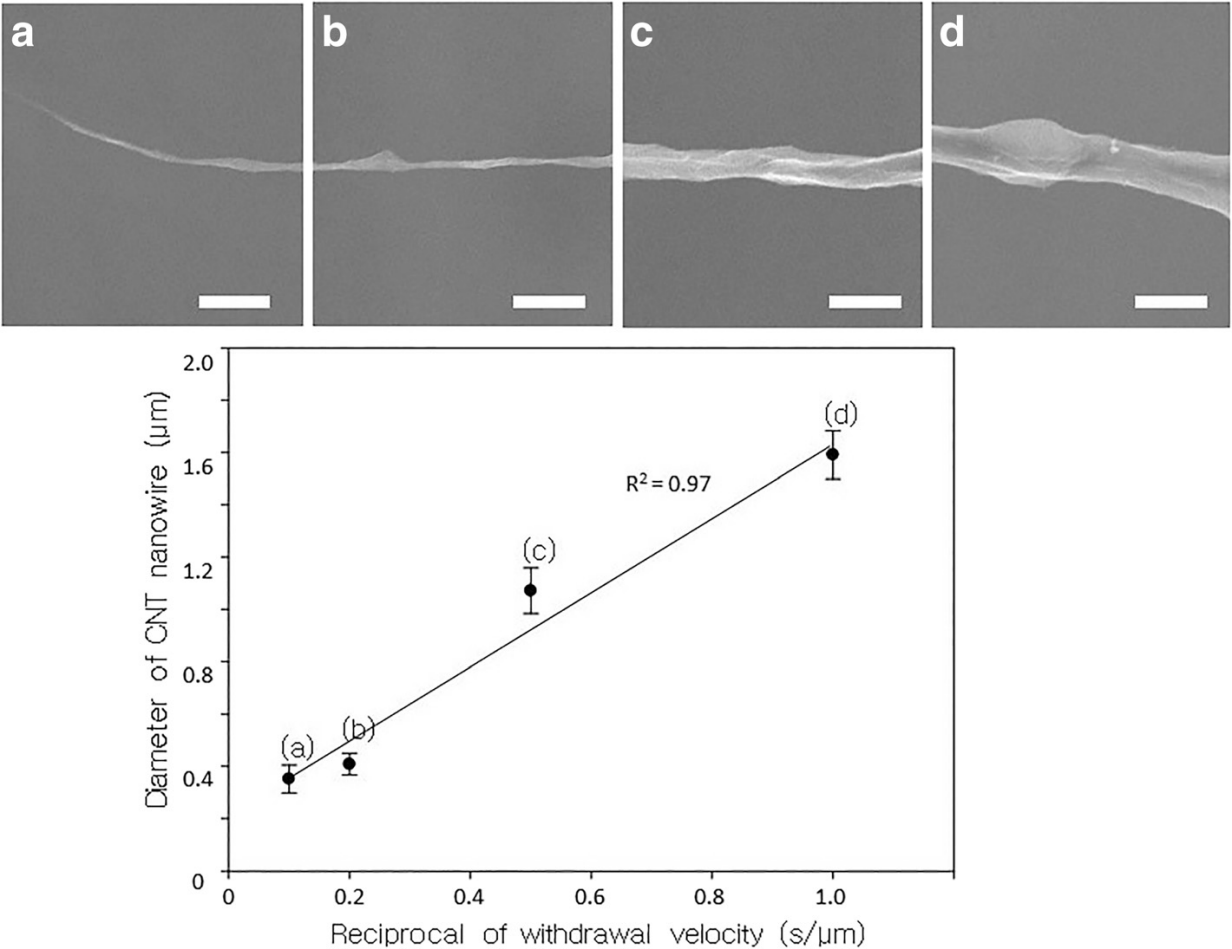
Scanning electron microscopy (SEM) images of CNT nanobundles with different diameters due to various withdrawal velocities. **a** *v*_w_ = 1  μm/s. **b** *v*_w_ = 2  μm/s. **c** *v*_w_ = 5  μm/s. **d** *v*_w_ = 10  μm/s. All were fabricated under 5*V*_pp_ and 1 MHz AC electric field. (Scale bar: 3 μm). (Mean ± standard error, *n* = 5)

In the steady state, the amount of CNTs attached to the synthesized CNT nanobundle can be represented as *A*_c_ = *ηC*_0_*d*, where *A*_c_, *η*, *C*_0_, and *d* are the amount of attached CNTs, the proportional constant, a concentration of CNTs, and the diameter of the synthesized CNT nanobundle, respectively. Assuming that a CNT nanobundle has a perfectly cylindrical shape, the volume (V) per unit time of a fabricated CNT nanobundle is *V* = *πd*^2^*v*_w_/4, where *v*_w_ is the withdrawal velocity of the tungsten tip. The volume per unit time can be expressed as *V* = *vA*_c_, where *v* is the volume of a single CNT. Finally, we can obtain the relationship of *dv*_w_ = const, because *v*, *C*_0_, and *η* are constant. This relationship means that the CNT nanobundle diameter is inversely proportional to the withdrawal velocity. Figures 5 and 6 show the experimental results for fabricating CNT nanobundles with four different withdrawal velocities of the tungsten tip under the same conditions (5 *V*_pp_ and 1 MHz AC electric field). The diameter of the CNT nanobundles decreased with increasing withdrawal velocity. These experimental results demonstrate that the diameter of the CNT nanobundles and the reciprocal of the withdrawal velocity had a linear proportional relationship. And the length of CNT nanobundle is mostly determined by an AC electrical turn on/off. When the electric filed is continuously applied, the length could reach to ~cm scale.

## Conclusions

In conclusion, a non-uniform electric field (positive DEP) was used to manipulate CNTs on a tungsten tip to fabricate a high-aspect-ratio CNT nanobundle. The withdrawal velocity of the tungsten tip was controlled automatically and precisely using a 1-D motorized stage. When the tungsten tip was pulled away from liquid–air interface, the capillary and van der Waals forces determined the diameter of the CNT nanobundle. Most of the CNTs attached to an electrode can be used to constitute a CNT nanobundle, because the capillary force acting on the CNTs is much smaller than the van der Walls force between the CNTs. It was determined that the withdrawal velocity of the tungsten tip was inversely proportional to the CNT nanobundle diameter, based on attachment efficiency. Finally, this was confirmed by experimentally controlling the withdrawal velocity from 1 to 10 μm/s under a constant AC electric field. We anticipate that the control technique using the withdrawal velocity could provide an important method for fabricating CNT nanobundles.
